# A pilot trial to evaluate the acute toxicity and feasibility of tamoxifen for prevention of breast cancer.

**DOI:** 10.1038/bjc.1989.235

**Published:** 1989-07

**Authors:** T. J. Powles, J. R. Hardy, S. E. Ashley, G. M. Farrington, D. Cosgrove, J. B. Davey, M. Dowsett, J. A. McKinna, A. G. Nash, H. D. Sinnett

**Affiliations:** Royal Marsden Hospital, Sutton, Surrey, UK.

## Abstract

Epidemiological and experimental evidence indicates that oestrogens are involved in the carcinogenic promotion of human breast cancer. We have undertaken a pilot trial of tamoxifen, an anti-oestrogen, compared to placebo given to 200 women at a high risk of developing breast cancer. The results of this trial show that acute toxicity is low and that accrual and compliance are satisfactory. Furthermore, biochemical monitoring of lipids and clotting factors indicate that tamoxifen may reduce the risk of cardiovascular deaths. At this stage no untoward long-term risks have been identified, and it is therefore proposed that a large multicentre trial should be started.


Br. J. Cancer (1989), 60, 126-131 © The Macmillan Press Ltd., 1989

### A pilot trial to evaluate the acute toxicity and feasibility of tamoxifen for prevention of breast cancer

T.J. Powles, J.R. Hardy, S.E. Ashley, G.M. Farrington, D. Cosgrove, J.B. Davey, M. Dowsett, J.A. McKinna, A.G. Nash, H.D. Sinnett, C.R. Tillyer & J.G. Treleaven

*The Royal Marsden Hospital, Downs Road, Sutton, Surrey, UK and Fulham Road, London SW3 6JJ, UK.*

**Summary** Epidemiological and experimental evidence indicates that oestrogens are involved in the carcinogenic promotion of human breast cancer. We have undertaken a pilot trial of tamoxifen, an anti-oestrogen, compared to placebo given to 200 women at a high risk of developing breast cancer. The results of this trial show that acute toxicity is low and that accrual and compliance are satisfactory. Furthermore, biochemical monitoring of lipids and clotting factors indicate that tamoxifen may reduce the risk of cardiovascular deaths. At this stage no untoward long-term risks have been identified and it is therefore proposed that a large multicentre trial should be started.

Experimental evidence indicates that oestrogens are involved in carcinogenic promotion of mouse mammary tumours (Jordan *et al.*, 1980; Jordan 1981). In humans epidemiological evidence supports the hypothesis that this mechanism may be important in the promotion of breast cancer (Miller & Bulbrook, 1980). This raises the therapeutic possibility that endocrine intervention could prevent breast cancer, (Cusick *et al.*, 1986) but it seems unlikely that more extensive epidemiological studies will adequately test this hypothesis.

An alternative approach would be to undertake a clinical trial using an effective anti-oestrogenic intervention in high risk women. This would not only provide evidence as to whether anti-oestrogenic intervention could prevent breast cancer, but also give strong indications about mechanisms of carcinogenesis in human breast cancer.

The epidemiological studies of breast cancer incidence following the atomic bomb explosions in Japan in 1945 indicate that ovarian function is required for endocrine promotion of radiation induced breast cancers. The incidence of breast cancer did not start to increase until about 13 years after radiation (Tokunaga *et al.*, 1979) and continues to increase 30 years later (Tokunaga *et al.*, 1984). This would suggest that endocrine prevention trials would require long periods of follow-up before any beneficial effects could be detected.

The number of women required for a prevention trial will depend on the number of cancers which eventually develop in the trial population. This, in turn, is a function of the relative risk of women chosen, the duration of follow-up and the magnitude of the preventative effect. In order to detect a 75% reduction only 15 breast cancers need to develop in the test population, whereas 300 cancers would be required in order to detect a 25% reduction. For unselected women in the UK aged between 35 and 65, the risk of breast cancer is approximately 1.75 per 1,000 per year (Cancer Statistics, 1984) whereas women aged 50–60 with a first degree female family history of breast cancer have a risk of 5–6 per 1,000 per year. Of these high risk women, 5,000 will develop 250–300 cancers over a 10-year follow-up period.

Thus, in order to have a reasonable chance of detecting significant prevention by anti-oestrogenic intervention, approximately 5,000 high-risk women would be required, with a 10–20 year follow-up. Were compliance maintained at only 50%, four times this number of women would be needed. This requires that the anti-oestrogenic intervention be well tolerated and easy to administer and maintain.

There are various anti-oestrogenic options available. A low fat diet may reduce oestrogenic activity (Rose *et al.*, 1987) but strict dietary control is required and it is unlikely that this could be maintained for the time required for adequate protection. Non-randomised studies indicate that oophorectomy or ovarian irradiation will substantially reduce the incidence of breast cancer, the degree of protection being related to age at ablation (MacMahon & Feinlieb, 1960; Hirayama & Wynder, 1962). However, it is neither realistic nor ethical to propose a 5,000 women ovarian ablation trial. An attractive alternative is the use of a physiological antagonist of oestrogen, such as a progestin or androgen, perhaps in combination with oestrogen as an oral contraceptive or as hormone replacement therapy. However, there is doubt as to whether a progestin would protect against breast cancer (Key & Pike, 1988).

Tamoxifen, a relatively selective anti-oestrogen, is effective treatment for endocrine sensitive breast cancer with virtually no side-effects (Cole *et al.*, Ward, 1973). When given to women with primary breast cancer it delays relapse and prolongs survival (Scottish Breast Cancer Trials Report, 1987; Nato Report, 1980; CRC Report, 1988). It is also an effective treatment for primary breast cancer in some elderly patients (Bradbeer & Kyngdon, 1983). Tamoxifen has been used to treat breast cancer since 1969 and no untoward long-term side effects have been identified in humans. Furthermore, preliminary toxicity studies indicate that tamoxifen is not anti-oestrogenic on other important tissues in the body, such as bone (Fentiman *et al.*, 1988), where lack of oestrogen could promote osteoporosis. Mutagenicity studies have indicated that, like oestrogen, tamoxifen at high dosages can cause hepatocellular cancer in the rat (DeWaard & Wang, 1988). Tamoxifen is generally oestrogenic in the rat and this effect is similar to that seen with the oral contraceptive pill. Hepatocellular cancer has not been reported in women taking tamoxifen and it appears likely that this risk is very small (Fentiman & Powles, 1987). With regard to other tumours, experimental evidence indicates that tamoxifen may be oestrogenic on the endometrium (Gottardis & Robinson, 1988) with a potential associated risk of causing endometrial cancer (Hardell, 1988; Jordan, 1988).

Retrospective analysis of new primary cancers in women on adjuvant tamoxifen (40 mg $day^{-1}$) in Sweden indicate an increased risk of endometrial cancer (Fornander *et al.*, 1989), although this has not been confirmed in the Scottish trial giving adjuvant tamoxifen 20 mg $day^{-1}$ for 5 years (Steward & Knight, 1989). This may be related to the higher dosage of tamoxifen in the Swedish trial. Overall it seems that the ease of administration, low acute toxicity with prospects of high long-term compliance, and proven anti-growth properties on endocrine sensitive breast cancer makes tamoxifen an ideal agent for a clinical trial of endocrine prevention of breast cancer.

We have therefore started a pilot double blind clinical trial of tamoxifen versus placebo in women with a high risk of developing breast cancer, in order to assess the feasibility of a large prevention programme.

Received 16 January 1989, and accepted in revised form 31 March 1989.

### Trial design

This double blind, placebo-controlled feasibility trial was designed to accrue 200 well women with at least one first degree relative who had had breast cancer. The initial draft protocol was drawn up in July 1985 and the proposed plan was presented to hospital colleagues, local general practitioners, potential participating women, the Cancer Research Campaign and the British Breast Group. In October 1986, ethical clearance was granted by the Royal Marsden Hospital, requiring informed, written and witnessed consent. Women were prescribed 'tamoplac' 20 mg $day^{-1}$ and were randomised to receive tamoxifen 20 mg $day^{-1}$ or placebo.

Between October 1986 and June 1987, 124 women were randomised. Accrual was then stopped following a mutagenicity report by ICI of liver tumours occurring in rats given high oral doses of tamoxifen. After further consideration by our Ethics Committee, ethical clearance was reconfirmed. Accrual was recommenced in February 1988, with stricter eligibility criteria (age and family history) and more extensive informed consent. The total of 200 patients for this feasibility trial was reached by October 1988.

Now that recruitment to this trial is complete, this interim analysis reports our rate of accrual, compliance, toxicity and monitoring for the first 2 years in order to assess if there is sufficient information to justify a large multicentre trial.

### Participants and methods

All women attending a symptomatic Medical Breast Clinic (SBC) at the Royal Marsden Hospital, Sutton or a screening clinic in the Early Diagnostic Unit (EDU) at the Royal Marsden Hospital, Fulham Road, aged 36–65 years old, who were neither pregnant nor at risk of pregnancy, not taking oral contraceptives or hormone replacement therapy, and who had no clinical or radiological evidence of breast cancer, were considered eligible for this trial. All eligible women were required to have at least one, and after February 1988 two, first degree female relatives with breast cancer. They were required to be psychologically, physically and geographically suitable for long-term screening. After counselling, eligible women were offered participation in the trial, including regular screening and monitoring. For entry, informed, witnessed and written consent was required.

At the commencement of the trial, the women were clinically assessed for breast abnormalities, with mammography, breast ultrasound and needle aspiration cytology where indicated. Post-randomisation, the women were seen at 3 and 6 months and then every 6 months for clinical examination, assessment of compliance and acute toxicity. During the development of this feasibility trial further monitoring was incorporated into the protocol as detailed below.

When possible serum oestradiol (E2) and sex hormone binding globulin (SHBG) were measured pre-treatment and at 3, 6, 9, 12, 18 and 24 months, in peri, post-menopausal and post-hysterectomy women. Pre-menopausal women had luteal and follicular-phase bloods taken pre-treatment and repeated at the above intervals. Serum was stored for other hormone measurements at a later stage.

The clotting factors anti-thrombin 3 and fibrinogen were measured if possible every 6 months along with a fasting lipid profile of total cholesterol, HDL cholesterol and triglyceride.

Some women in this study reported lower abdominal pains possibly caused by the development of ovarian cysts. We therefore undertook sequential pelvic ultrasound on a subsection of women using an Acuson scanner.

The risk of causing or aggravating osteoporosis by anti-oestrogenic intervention indicated a need for sequential measurement of bone mass. Hormone dependent bone mass can be adequately monitored by single photon absorption through the forearm using a bone densitometer (ND 110 Nuclear Data Inc.) (Christiansan & Rodbro, 1977; Christiansan *et al.*, 1981). This was repeated at 6-monthly intervals for the first 2 years and will be continued annually.

It is planned to continue medication, monitoring and screening in these 200 women for as long as is acceptable in order to gain information regarding long-term compliance and toxicity.

### Results

Until the stricter consent criteria were instituted in February 1988, 76 eligible women with at least one first degree relative with breast cancer consented to enter the trial from the EDU compared to 50 women for the SBC. From February 1988 to October 1988, 61 women from the EDU consented compared to 13 from the SBC (Table I).

After the change in eligibility criteria and consent in February 1988, a subpopulation of 242 women attending the EDU with a family history of breast cancer were assessed for reasons of non-eligibility and non-consent (Table II). Although an average of 11.5 women with a family history of breast cancer attended the clinic only an average of 2.7 were eligible, 47% of whom consented to inclusion after informed consent. Details of the reasons for ineligibility and non-consent are given in Table II.

For all 200 women randomised the age, menopausal status and extent of family history were similar for the tamoxifen and placebo women (Table III). Compliance to medication for the first year, as assessed by interview, was high and the same for both groups. This reflects the relatively low toxicity for tamoxifen versus placebo (Table IV). Apart from hot flushes which occurred in 27% of tamoxifen women versus 11% of placebo women ($P<0.005$) there was no significant differences between the two types of medication.

Non-compliance related to toxicity was similar for both groups of women, with seven tamoxifen and six placebo women temporarily stopping medication. Permanent cessation of medication occurred in 18 tamoxifen versus 12 placebo women (n.s.) (Table V).

**Table I** Overall accrual rate of women for the Early Diagnostic Unit (EDU) and the Symptomatic Breast Clinic (SBC)

| | *EDU* | *SBC* | *Total* |
|---|---|---|---|
| Oct. to Dec. 1986 | 5 | 16 | 21 |
| Jan. to Mar. 1987 | 28 | 17 | 45 |
| Apr. to June 1987 | 42 | 16 | 58 |
| July to Sep. 1987 | 1 | 0 | 1 |
| Oct. to Dec. 1987 | 0 | 1 | 1 |
| Jan. to Mar. 1988 | 10 | 4 | 14 |
| Apr. to June 1988 | 13 | 5 | 18 |
| July to Sep. 1988 | 38 | 4 | 42 |

**Table II** Eligibility, consent and accrual rates into the tamoxifen prevention trial for the sample population attending 21 screening sessions in the EDU between February and July 1988

| | | |
|---|---|---|
| No. of women with FH | 242 | 11.5/clinic |
| No. eligible | 62 (26%) | 2.95/clinic |
| Ineligible | 180 | |
| Age 40–60 years | 65 (36%) | |
| Pregnancy risk | 89 (49%) | |
| Family history <1 relative | 75 (42%) | |
| Pill or HRT | 22 (12%) | |
| General medical | 20 (11%) | |
| Carcinoma on screen | 1 (0.4%) | |
| No. consenting (% of eligible) | 29 (47%) | 1.4/clinic |
| Non-consent | 33 | |
| Geographical | 10 | |
| Not acceptable | 24 | |

**Table III** Characteristics of 100 women randomised to tamoxifen compared to 100 women randomised to placebo

| | *Tamoxifen* (*100*) | *Placebo* (*100*) | |
|---|---|---|---|
| Age, mean years (range) | 48 (31–66) | 49 (30–64) | |
| Family history of 1st degree relative | | | |
| 0 | 2 | 0 | n.s. |
| 1 | 38 | 47 | |
| 2 | 35 | 36 | |
| >2 | 24 | 14 | |
| Premenopausal | 58 | 52 | n.s. |
| Perimenopausal (<2 years LMP) | 2 | 1 | |
| Post-menopausal | 23 | 24 | |
| Post-hysterectomy | 15 | 21 | |
| Compliance | | | |
| 3 months | 89% | 94% | n.s. |
| 6 months | 85% | 91% | |
| 9 months | 83% | 88% | |
| 12 months | 83% | 85% | |

**Table IV** Number (%) of women with acute toxicity, symptomatic effects and compliance for 75 women on tamoxifen compared to 75 women on placebo, who had been on medication for at least 3 months

| | *Tamoxifen* *No. (%)* | *Placebo* *No. (%)* | |
|---|---|---|---|
| *Side-effects* | | | |
| Nausea/sickness | 6 (8) | 11 (15) | n.s. |
| Headaches | 7 (9) | 5 (7) | n.s. |
| Hot flushes | 20 (27) | 8 (11) | $P<0.005$ |
| Amenorrhoea | 10 (13) | 9 (12) | |
| Vaginal discharge | 4 (5) | 0 | |
| Weight gain (>2 kg) | 3 (4) | 3 | |
| Muscle cramps | 4 (5) | 0 | |
| Depression | 1 (1) | 0 | |
| Abdominal pains | 2 (3) | 0 | |
| *Symptomatic relief* | | | |
| Breast pain | 8/13 | 10/21 | |
| Breast nodules | 22/52 | 17/50 | |
| Premenstrual | | | |
| Breast symptoms | 18/24 | 17/24 | |
| Fluid retention | 9/19 | 6/18 | |
| Headaches | 8/13 | 4/13 | |
| Tension | 7/12 | 2/8 | |

**Table V** Non-compliance of tamoxifen/placebo medication

| | *Tamoxifen* | *Placebo* | |
|---|---|---|---|
| *Temporary cessation* | 8 | 7 | n.s. |
| Non-toxic | 1 | 1 | |
| Toxic | 7 | 6 | |
| Nausea | 1 | 1 | |
| Headaches | 1 | 2 | |
| Hot flushes | 0 | 1 | |
| Weight gain | 0 | 2 | |
| Irregular periods | 1 | 0 | |
| Vaginal discharge | 1 | 0 | |
| Other | 3 | 0 | |
| *Permanent cessation* | 18 | 12 | n.s. |
| Non-toxic | 6[a] | 6 | n.s. |
| Toxic | 12 | 6 | |
| Nausea | 3 | 2 | |
| Vomiting | 1 | 1 | |
| Headaches | 1 | 1 | |
| Hot flushes | 4 | 1 | |
| Weight gain | 1 | 0 | |
| Irregular periods | 0 | 2 | |
| Abdominal pain | 1 | 0 | |
| Vaginal discharge | 1 | 0 | |
| Other | 4 | 3 | |

[a]Including two non-starters.

Ovarian ultrasound was carried out in 19 women before the start of medication and 38 women up to one year later (Table VI). Five (25%) of the 20 women on tamoxifen had at least one ovarian cyst identified compared to two of 19 pre-treatment and none of 18 post-placebo women (i.e. a total of two cysts (4%) in 37 non-tamoxifen women). Sequential pelvic ultrasound studies continue on as many women as possible in order to assess the clinical significance of these findings.

Clotting studies were undertaken in as many women as possible at various times during the trial, measuring levels of fibrinogen and anti-thrombin 3 (Figure 1). There was a non-significant trend towards lower levels of fibrinogen, and, to a lesser extent, anti-thrombin 3 for women on tamoxifen.

**Table VI** Ovarian ultrasound (Acuson Scanner) on 55 women, 19 pre-medication, 20 post-medication on tamoxifen and 18 post-medication on placebo (two women had sequential studies)

| | *Pre-treatment* (*19*) | *Post-treatment* (*38*) | |
|---|---|---|---|
| *Tamoxifen* | 10 | 20 | |
| Normal | 7 | 11 | |
| Cyst | 1 | 5 (25%) | n.s. |
| Fibroids | 2 | 4 | |
| *Placebo* | 9 | 18 | |
| Normal | 8 | 16 | |
| Cysts | 1 | 0 | |
| Fibroids | 1 | 2 | |

**Figure 1** Plasma levels of fibrinogen, anti-thrombin 3 (AT3) and ratio of fibrinogen/AT3 in women receiving tamoxifen (Tam, ●——●), or placebo (Plac, ○——○). The number of women from whom measurements were made at various times after randomisation are shown.

There was also a trend for a lower fibrinogen/anti-thrombin 3 ratio, which would possibly favour protection against pathological thrombosis. Most women in the trial are now included in these studies.

For logistic reasons it was initially difficult to organise a system for measurement of fasting lipids. However, this is now underway and women have fasting blood samples taken whenever possible before commencement of medication. Although the information to date shows no differences in triglycerides, HDL cholesterol or uric acid, levels of total cholesterol are significantly lower for women on tamoxifen (Figure 2). Our normal total cholesterol ranges from 3.6 to 6.8 mmol $l^{-1}$. For sequential measurements in 49 women the mean change in plasma cholesterol levels on placebo was $-0.08$ mmol $l^{-1}$ (95% confidence limits $+0.27$ to $-0.42$) compared to $-0.85$ mmol $l^{-1}$ (95% confidence limits $-0.45$ to $-1.25$) ($P=0.02$) for those on tamoxifen.

Measurement of bone mineral content showed no evidence of increased bone mineral loss for women on tamoxifen (Figure 3). Furthermore, tamoxifen had no effect on bone mineral loss in pre-menopausal compared to post-menopausal women.

We have undertaken preliminary measurements of serum E2 and SHBG in some women. There was no difference in pre-treatment and post-treatment SHBG levels in 20 women who received placebo (mean $-2.6$ nmol $l^{-1}$, 95% CI, $+5$ nmol $l^{-1}$ to $-10$ nmol $l^{-1}$) compared to a significant increase for women on tamoxifen (mean $+14$ nmol $l^{-1}$, 95% CI, $+21$ nmol $l^{-1}$ to $+8$ nmol $l^{-1}$, $P<0.001$).

**Figure 2** The plasma levels of total cholesterol in women receiving tamoxifen (Tam, ●——●) or placebo (Plac, ○——○). The number of women for whom measurement was made at various times after randomisation are shown.

**Figure 3** Age corrected bone mineral content shown as % of estimated normal measurement using a single photon forearm bone densitometer. Tamoxifen (Tam, ●——●) or placebo (Plac, ○——○).

**Figure 4** Mean differences between pre- and on-treatment levels with 95% confidence intervals for tamoxifen (●——●) and placebo (○——○) in pre-menopausal (luteal) and post-menopausal oestradiol (E2) and sex hormone binding globulin SHBG.

There was no difference in pre- and on-treatment levels of serum oestradiol for 14 post-menopausal women on placebo (mean $-8$ pmol $l^{-1}$ 95% CI $+7$ pmol $l^{-1}$ to $-23$ pmol $l^{-1}$) or tamoxifen (mean $+5$ pmol $l^{-1}$ 95% CI, $+50$ pmol $l^{-1}$ to $-40$ pmol $l^{-1}$). In contrast there was a significant increase in on-treatment levels of serum oestradiol for 16 pre-menopausal women who received either tamoxifen (mean $+700$ pmol $l^{-1}$, 95% CI, $+1040$ pmol $l^{-1}$ to $+360$, $P<0.001$) or placebo (mean $+262$ pmol $l^{-1}$, 95% CI $+648$ pmol $l^{-1}$ to $+55$ pmol $l^{-1}$, $P<0.005$).

These results confirm previous reports that tamoxifen causes a rise in the serum levels of E2 in pre-menopausal women (Jordan, 1986) and SHBG in pre- and post-menopausal women (Sakai *et al.*, 1978). Further analyses of sequential changes in other hormones are underway.

### Discussion

The aim of this trial was to test the feasibility of a large multicentre prevention trial using tamoxifen in high risk women. From two breast clinics we were able to identify about seven eligible women per week and after full discussion approximately 50% of them consented to randomisation into the trial, in spite of extensive consent and ethical requirements for this trial. More eligible women were identified and there was a higher acceptance rate in the screening clinic, compared to the symptomatic breast clinic. This presumably reflects an increased interest in early diagnosis and prevention in this clinic.

Generally, there was little difference in acute toxicity between tamoxifen and placebo. Hot flushes occurred in significantly more women on tamoxifen but generally were only mild. Menstrual changes including amenorrhoea were not symptomatically a problem and relief of pre-menstrual tension and headaches were of benefit to some patients on tamoxifen. Cessation of treatment because of toxicity was similar for tamoxifen and placebo. Compliance was the same for tamoxifen and placebo and was surprisingly high, with greater than 80% of women continuing medication after the first year. This relates to the ease of medication and low toxicity and may approach the maximum achievable compliance with any endocrine intervention.

With regard to long-term toxicity we are, at present unable to identify any changes which might indicate future potential risks. Changes in lipids and SHBG indicate a similar oestrogenic effect on the liver to those reported with oral contraceptives or hormone replacement therapy (Sakai *et al.*, 1978; Rossner & Wallgren, 1984). The effects that these changes may have on the incidence of coronary heart disease or atherosclerosis are difficult to predict. It would seem that a rise in HDL cholesterol and a lowering in LDL cholesterol and thereby total cholesterol is beneficial (Bush & Miller, 1987; Bush & Barratt-Connor, 1985). We are therefore undertaking more extensive lipid studies to answer these questions.

Changes in clotting factors with a lowering of the fibrinogen/anti-thrombin 3 ratio may indicate a decreased risk of thrombosis, especially if this is further enhanced by a significant reduction in serum cholesterol. This may account for an apparent decrease in non-cancer deaths in two major adjuvant trials (Scottish Trial Report, 1987; Nato Report, 1980).

Sequential pelvic ultrasound examination in a small subgroup of women failed to detect a significant effect of tamoxifen on the ovaries or uterus. At this stage it would appear that any possible increased risk of ovarian or uterine cancer is outweighed by a potential reduced risk of cardiovascular disease. It is possible that a long-term, unexpected and serious toxicity of tamoxifen may be identified in the future, but at this stage it would seem that this is small compared with the probability of developing breast cancer in these high risk women. It is anticipated that regular breast, ovarian, uterine, lipid and clotting screening, together with a possible reduction in cardiovascular deaths, would more than outweigh any potential unknown risks.

The results from this feasibility trial indicate that it would be possible to accrue sufficient high risk women into a national programme. This would be assisted by identification of high risk eligible women within the national screening programme. Accrual of 5,000 women with at least two first degrees female relatives could probably be achieved within 5 years. Using tamoxifen or placebo, adequate compliance could be maintained in order to detect a 25% prevention effect at 10–15 years.

Problems of work load and cost are likely to be significant. The cost of tamoxifen and placebo for the feasibility trial is approximately thirty pounds per woman, per year of medication. The cost of active screening of 5,000 women, together with the added extra supervision required for medication and toxicity assessment, would need to be considered within a national programme of screening and prevention.

In conclusion, these preliminary results indicate that tamoxifen, a proven anti-proliferative agent for endocrine sensitive breast cancer, is, for the most part, not anti-oestrogenic in other tissues of the body. This selective activity, with possible long-term benefits for bone and vascular disease, together with its low acute toxicity, makes it an ideal agent for a trial of endocrine prevention of breast cancer.

Further progress on the developments of a national multicentre trial will now depend on extended ethical approval and the support of such bodies as the Breast Cancer Trials Co-ordinating Sub-committee and the Cancer Research Campaign, or other national cancer research bodies.

We thank Farmitalia Carlo Erba for the supply of tamoxifen and placebo at cost. Without their help this feasibility trial would not have been possible.

### References

ANALYSIS OF CRC ADJUVANT BREAST TRIAL (1988). Cyclophosphamide and tamoxifen as adjuvant therapies in the management of breast cancer. *Br. J. Cancer*, **57**, 604.

BRADBEER, J.W. & KYNGDON, J. (1983). Primary treatment of breast cancer in elderly women with tamoxifen. *Clin. Oncol.*, **9**, 31.

BUSH, T.L. & BARRAT-CONNOR, E. (1985). Non-contraceptive oestrogen use and cardiovascular disease. *Epidemiol. Rev.*, **4**, 80.

BUSH, T.L. & MILLER, V.T. (1987). Effects of pharmacological agents used during menopause: impact on lipids and lipoproteins. In *Menopause: Physiology and Pharmacology*, Mishell, D.R. (ed) p. 187. Year Book Med. Publ.: Chicago.

CANCER STATISTICS REGISTRATION (1984). *Series MBI, vol. 16*. HMSO: London.

CHRISTIANSAN, C., CHRISTIANSAN, M.S. & TRANSBOL, I. (1981). Bone mass in post-menopausal women after withdrawal of oestrogen/gestagen replacement therapy. *Lancet*, **i**, 459.

CHRISTIANSAN, C. & RODBRO, P. (1977). Long-term reproducibility of bone mineral content measurements. *Scand. J. Clin. Lab. Invest.*, **37**, 321.

COLE, M.P. JONES, C.T.A. & TODD, I.D.H. (1971). A new anti-oestrogenic agent in late breast cancer. An early clinical appraisal of ICI 46474. *Br. J. Cancer*, **25**, 270.

CUSICK, J., WANG, D.Y. & BULBROOK, R.D. (1986). The prevention of breast cancer. *Lancet*, **i**, 83.

DE WAARD, F. & WANG, D.Y. (1988). Epidemiology and prevention: workshop report. *Eur. J. Cancer Clin. Oncol.*, **1**, 45.

FENTIMAN, I.S., CALEFFI, M., MURBY, B. & FOGELMAN, I. (1988). Dosage, duration and short term-effect on bone mineral content of Tamoxifen treatment for mastalgia. *Br. J. Clin. Prac.*, **52**, suppl. 56, 18.

FENTIMAN, I.S. & POWLES, T.J. (1987). Tamoxifen and benign breast problems. *Lancet*, **ii**, 1070.

FORNANDER, T., RUTQVIST, L.E., CEDERMARK, B. and 9 others (1989). Adjuvant Tamoxifen in early breast cancer: occurrence of new primary cancers. *Lancet*, **i**, 117.

GOTTARDIS, M.M. & ROBINSON, S.P. (1988). Contrasting actions of tamoxifen on endometrial and breast tumour growth in the athymic mouse. *Cancer Res.*, **48**, 812.

HARDELL, L. (1988). Tamoxifen as risk factor for carcinoma of corpus uteri. *Lancet*, **ii**, 563.

HIRAYAMA, T. & WYNDER, E.L. (1962). Study of epidemiology of cancer of the breast. Influence in hysterectomy. *Cancer*, **15**, 28.

JORDAN, V.C. (1981). Comparative anti-oestrogen action in experimental breast cancer. *Adv. Exp. Med. Biol.*, **138**, 165.

JORDAN, V.C., FRITZ, N.F. & VAN BEURDEN, M. (1986). Prophylactic tamoxifen (letter). *Lancet*, **i**, 910.

JORDAN, V.C. NAYLOR, K.E., DIX, C.J. & PRESTWICH, G. (1980). Anti-oestrogen action in experimental breast cancer. *Recent Results Cancer Res.*, **10**, 34.

JORDAN, V.C. (1988). Tamoxifen and endometrial cancer. *Lancet*, **ii**, 1019.

KEY, T.J.A. & PIKE, M.C. (1988). The role of oestrogens and progestagens in the epidemiology and prevention of breast cancer. *Eur. J. Clin. Oncol.*, **24**, 29.

MACMAHON, B. & FEINLIEB, M. (1960). Breast cancer in relation to nursing and menopausal history. *J. Natl Cancer Inst.*, **24**, 733.

MILLAR, A.B. & BULBROOK, R.D. (1980). The epidemiology and etiology of breast cancer. *N. Engl. J. Med.*, **303**, 1246.

NATO REPORT (1980). Controlled trial of tamoxifen as a single adjuvant agent in the management of early breast cancer. Analysis at 8 years. *Br. J. Cancer*, **57**, 608.

REPORT FROM THE SCOTTISH BREAST CANCER TRIALS (1987). Adjuvant tamoxifen in the management of operable breast cancer. The Scottish Trial. *Lancet*, **ii**, 172.

ROSE, D.P., BOYAR, A.P., COHEN, C. & STRONG, L.E. (1987). The effect of low fat diet on hormone levels in women with cystic breast disease. *J. Natl Cancer Inst.*, **78**, 623.

ROSSNER, S. & WALLGREN, A. (1984). Serum lipoproteins after breast cancer surgery and effects of tamoxifen. *Atherosclerosis*, **52**, 339.

SAKAI, F., CHEIX, F., CLAVEL, M. *et al.* (1978). Increase in steroid binding globulins induced by tamoxifen in patients with breast cancer. *J. Endocrinol.*, **76**, 219.

STEWARD, H.J. & KNIGHT, G.M. (1983). Tamoxifen and the uterus and endometrium. *Lancet*, **i**, 375.

TOKUNAGA, M., LAND, C.E., YAMAMATO, T. & 5 others (1984). Breast cancer among atomic bomb survivors. In *Radiation Carcinogenesis: Epidemiology and Biological Significance*, Boice, J.D. & Franmeni, J.F. (eds) Raven Press: New York.

TOKUNAGA, M., NORMAN, J.E., ASANO, M. *et al.* (1979). Maliguant breast tumours among atomic bomb survivors, Hiroshima and Nagasaki, 1959–74. *J. Natl Cancer Inst.*, **62**, 1347.

WARD, H.W.C. (1973). Anti-oestrogen therapy for breast cancer. A trial of tamoxifen at low dose levels. *Br. Med. J.*, **i**, 13.


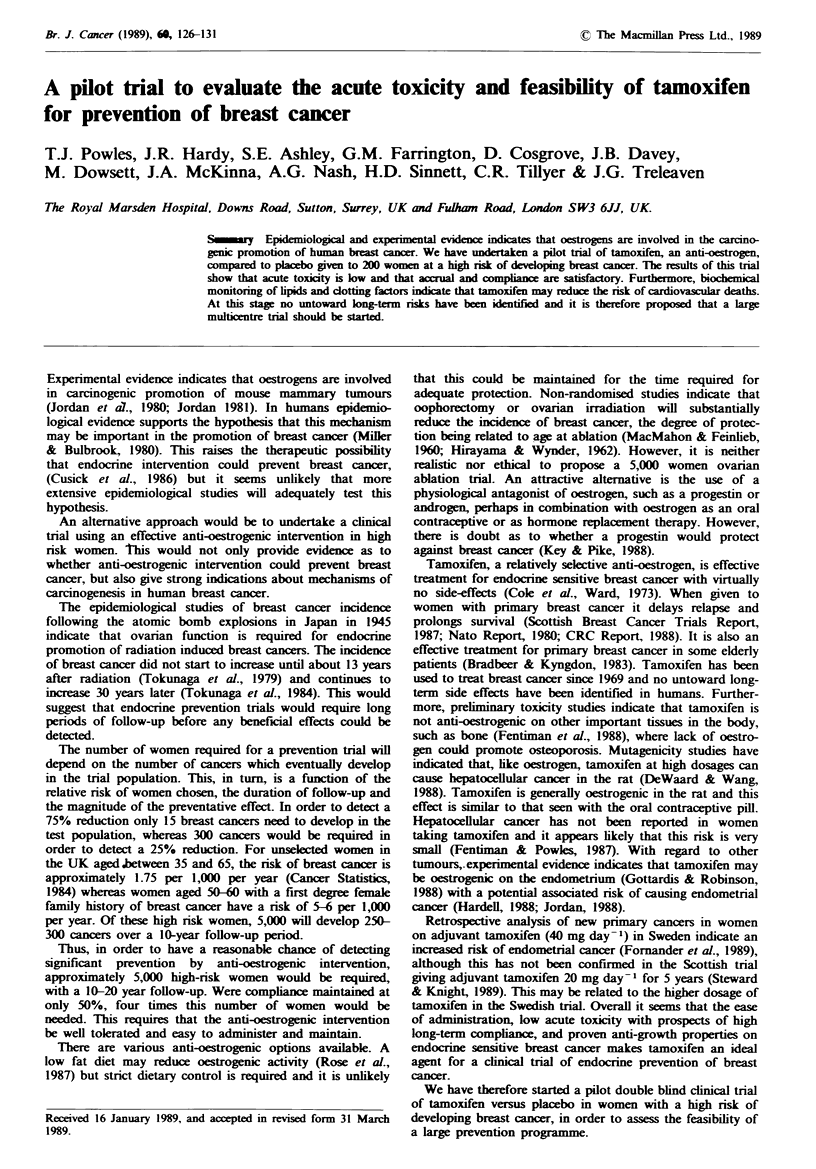

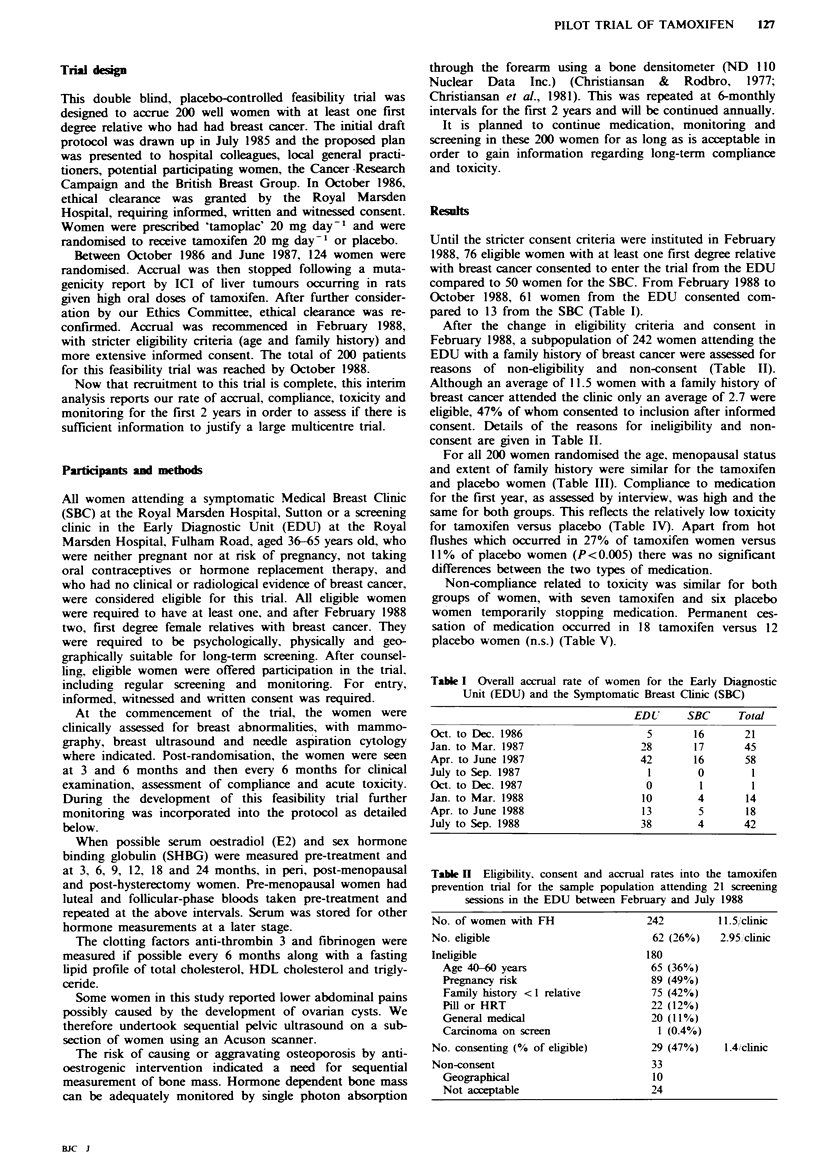

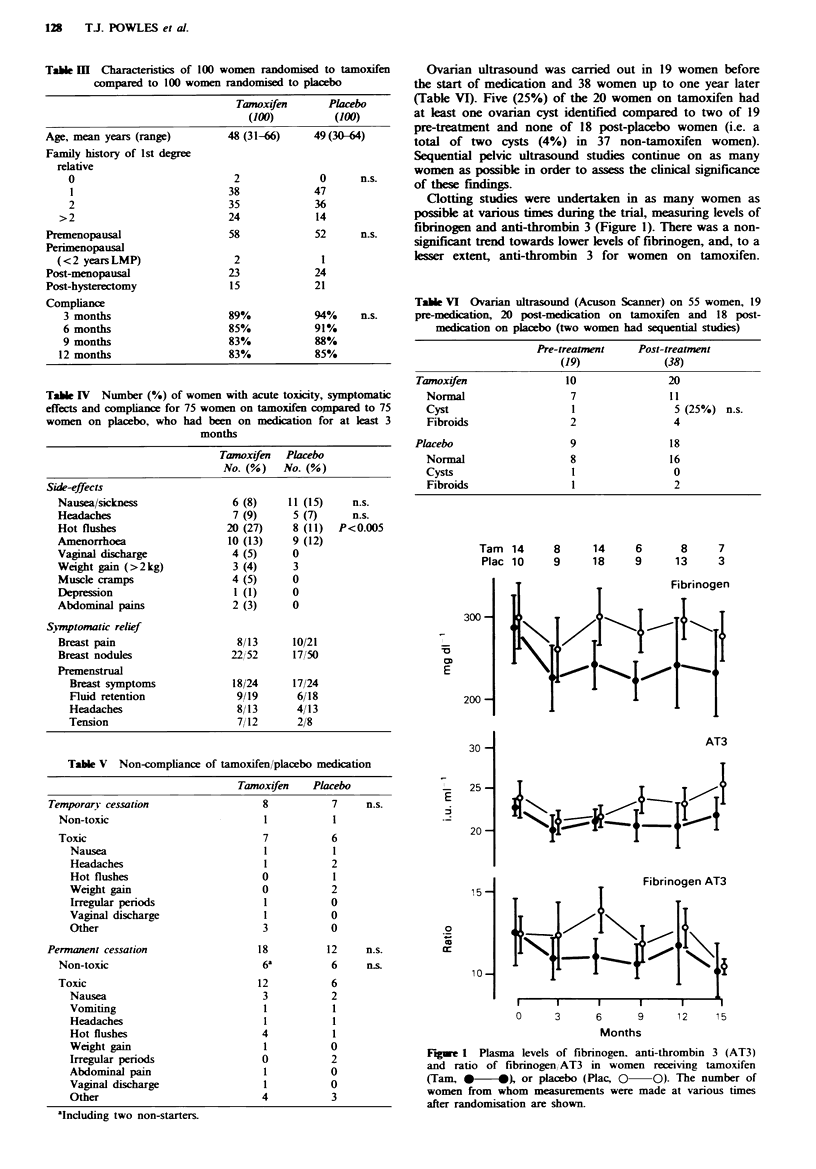

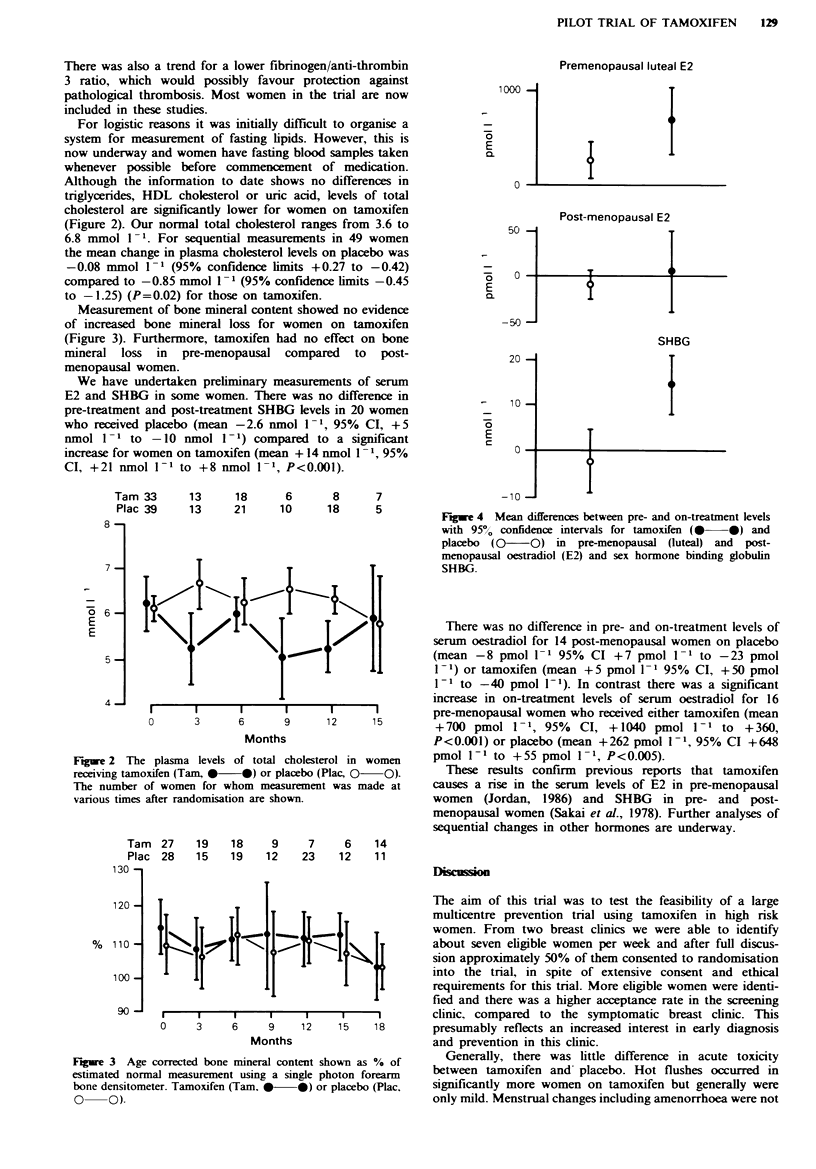

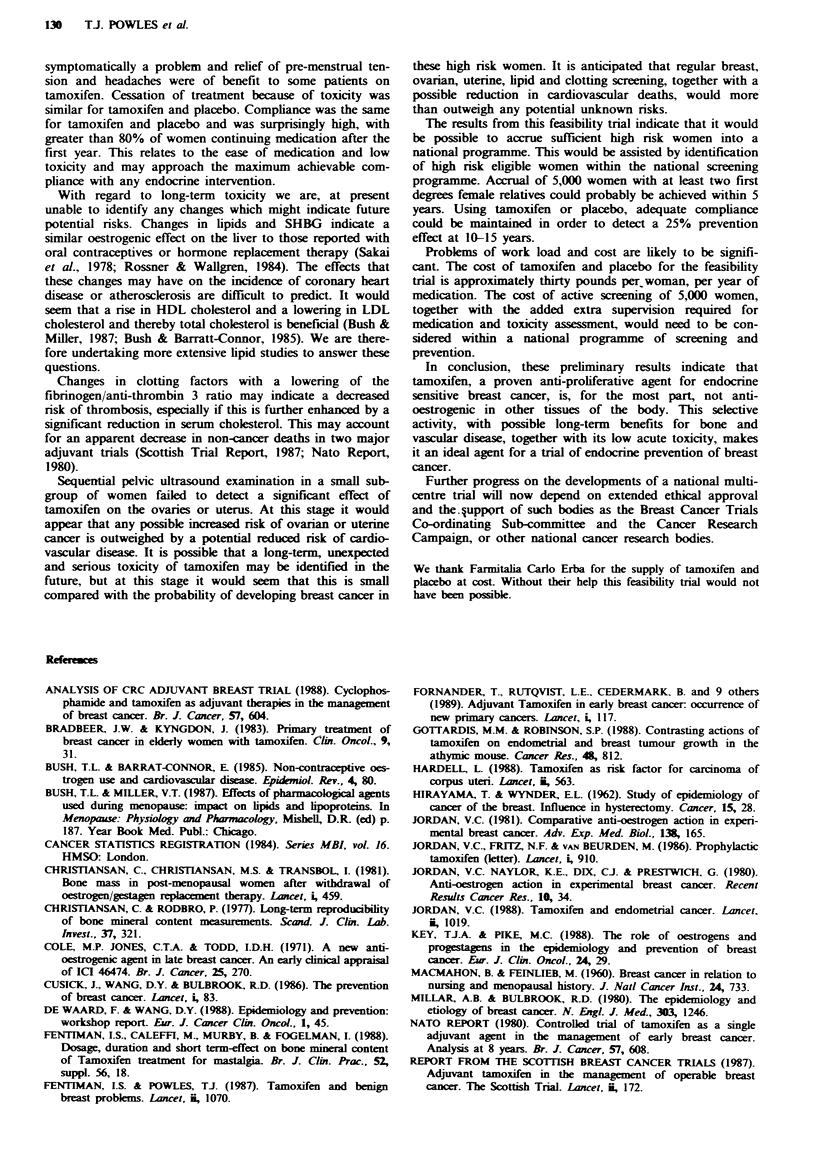

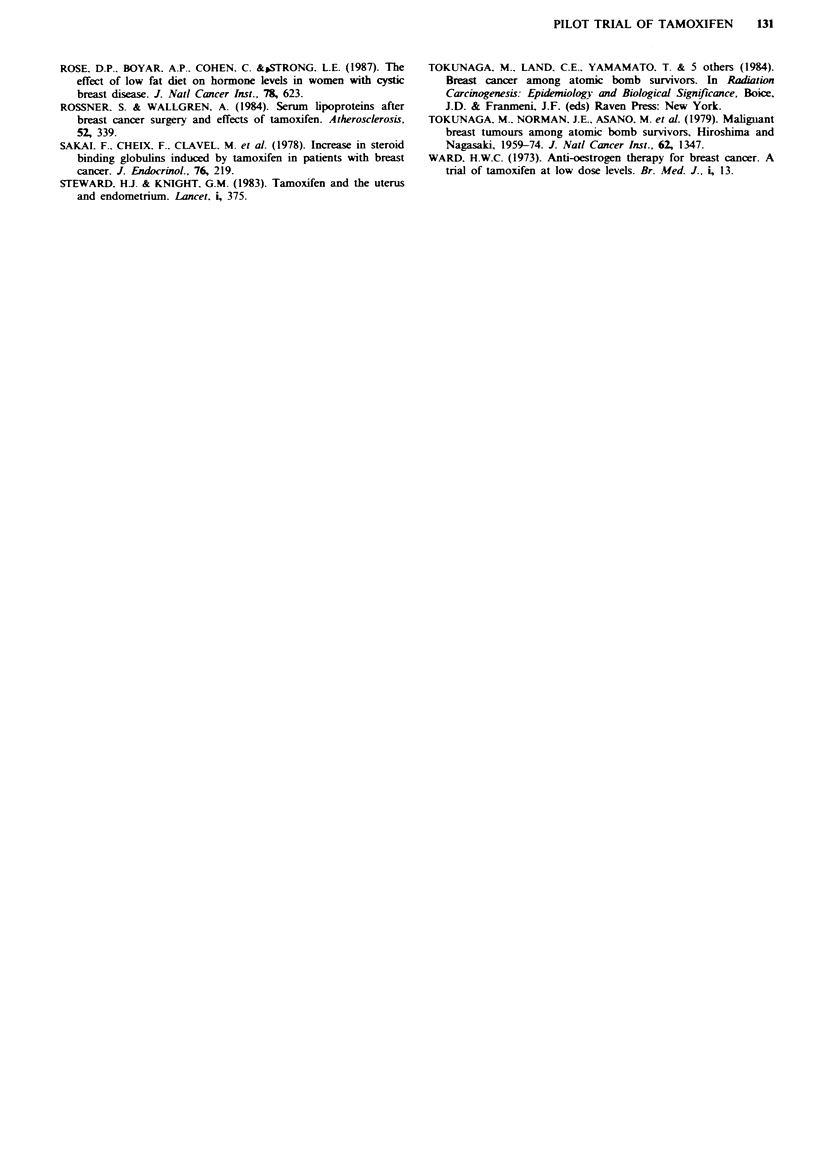


## References

[OCR_00827] Bradbeer J. W., Kyngdon J. (1983). Primary treatment of breast cancer in elderly women with Tamoxifen.. Clin Oncol.

[OCR_00846] Christiansen C., Christensen M. S., Transbøl I. (1981). Bone mass in postmenopausal women after withdrawal of oestrogen/gestagen replacement therapy.. Lancet.

[OCR_00851] Christiansen C., Rödbro P. (1977). Long-term reproducibility of bone mineral content measurements.. Scand J Clin Lab Invest.

[OCR_00856] Cole M. P., Jones C. T., Todd I. D. (1971). A new anti-oestrogenic agent in late breast cancer. An early clinical appraisal of ICI46474.. Br J Cancer.

[OCR_00865] De Waard F., Wang D. Y. (1988). Epidemiology and prevention: workshop report.. Eur J Cancer Clin Oncol.

[OCR_00875] Fentiman I. S., Powles T. J. (1987). Tamoxifen and benign breast problems.. Lancet.

[OCR_00879] Fornander T., Rutqvist L. E., Cedermark B., Glas U., Mattsson A., Silfverswärd C., Skoog L., Somell A., Theve T., Wilking N. (1989). Adjuvant tamoxifen in early breast cancer: occurrence of new primary cancers.. Lancet.

[OCR_00884] Gottardis M. M., Robinson S. P., Satyaswaroop P. G., Jordan V. C. (1988). Contrasting actions of tamoxifen on endometrial and breast tumor growth in the athymic mouse.. Cancer Res.

[OCR_00889] Hardell L. (1988). Tamoxifen as risk factor for carcinoma of corpus uteri.. Lancet.

[OCR_00896] Jordan V. C. (1981). Comparative antioestrogen action in experimental breast cancer.. Adv Exp Med Biol.

[OCR_00900] Jordan V. C., Fritz N. F., van Beurden M. (1986). Prophylactic tamoxifen.. Lancet.

[OCR_00909] Jordan V. C. (1988). Tamoxifen and endometrial cancer.. Lancet.

[OCR_00918] MACMAHON B., FEINLEIB M. (1960). Breast cancer in relation to nursing and menopausal history.. J Natl Cancer Inst.

[OCR_00922] Miller A. B., Bulbrook R. D. (1980). The epidemiology and etiology of breast cancer.. N Engl J Med.

[OCR_00938] Rose D. P., Boyar A. P., Cohen C., Strong L. E. (1987). Effect of a low-fat diet on hormone levels in women with cystic breast disease. I. Serum steroids and gonadotropins.. J Natl Cancer Inst.

[OCR_00943] Rössner S., Wallgren A. (1984). Serum lipoproteins and proteins after breast cancer surgery and effects of tamoxifen.. Atherosclerosis.

[OCR_00948] Sakai F., Cheix F., Clavel M., Colon J., Mayer M., Pommatau E., Saez S. (1978). Increases in steroid binding globulins induced by tamoxifen in patients with carcinoma of the breast.. J Endocrinol.

[OCR_00963] Tokunaga M., Norman J. E., Asano M., Tokuoka S., Ezaki H., Nishimori I., Tsuji Y. (1979). Malignant breast tumors among atomic bomb survivors, Hiroshima and Nagasaki, 1950-74.. J Natl Cancer Inst.

